# Prognostic Significance of B-Type Natriuretic Peptide in Patients With Left Ventricular Thrombus

**DOI:** 10.3389/fcvm.2021.667908

**Published:** 2021-04-29

**Authors:** Zhixia An, Zhichun Gao, Luyu Wang, Changchun Hou, Liying Zhang, Siming Gong, Rongsheng Rao, Chun Li, Zhexue Qin

**Affiliations:** ^1^Department of Cardiology, Xinqiao Hospital, Army Medical University (Third Military Medical University), Chongqing, China; ^2^Institute of Cardiovascular Diseases of PLA, Xinqiao Hospital, Army Medical University (Third Military Medical University), Chongqing, China; ^3^Department of Cardiology, Shapingba District People's Hospital, Chongqing, China; ^4^Department of Ultrasound, Xinqiao Hospital, Army Medical University (Third Military Medical University), Chongqing, China

**Keywords:** B-type natriuretic peptide, ventricular thrombus, mortality risk, prognostic, systemic embolism

## Abstract

**Background and Aims:** There is sparse information on the prognostic value of B-type natriuretic peptide (BNP) for the outcomes in patients with left ventricular thrombus (LVT).

**Methods:** Patients diagnosed with LVT by transthoracic echocardiography between November 2009 to July 2020 at our institution were included. The endpoints were all-cause mortality and systemic embolism.

**Results:** Ninety-two subjects were finally included in the study. The mean age of the cohort was 56.73 ± 14.12, and 80.4% of the patients were male. The median BNP (1st quartile−3rd quartile) was 437.5 (112.74–1317.5). The total all-cause mortality rate was 30.44% (28/92), and the 1-year, 2-year, and 3-year cumulative survival rates were 85.4, 75.5, and 66.5%, respectively. Systemic embolism was identified in 10 subjects. COX multivariate analysis showed that Log BNP (HR, 4.16; 95%CI, 1.81–9.56; *P* = 0.001) and BMI (HR, 0.86; 95%CI, 0.73–0.99; *P* = 0.048) were significantly associated with all-cause mortality. In addition, patients with BNP levels in the upper median (≥ 437.5 pg/ml) had significantly higher all-cause mortality rate compared to those with lower median BNP (<437.5 pg/ml; *P* = 0.004). The area under the receiver operating characteristic curve for BNP and all-cause mortality was 0.71. In the linear trend test, BNP quartiles were significantly related to all-cause mortality in all models, and the *P*-values for trend in models 1, 2, and 3 were 0.005, 0.006, and 0.048, respectively.

**Conclusion:** BNP level is a prognostic factor for all-cause mortality in LVT patients, and elevated BNP is indicative of a higher risk of LVT.

## Introduction

Left ventricular thrombus (LVT) is a complication of systolic dysfunction, and usually afflicts patients with myocardial infarction or dilated cardiomyopathy. Both ischemic and non-ischemic LVT formation follow the Virchow's triad including reduced ventricular wall motion, endomyocardial injury and hypercoagulability/stasis of blood flow (1, 2). Despite primary percutaneous coronary intervention (PCI) and the significant scientific breakthrough beyond foundational neurohormonal therapies improving the prognosis of heart failure (HF) in recent decades, patients with LVT are at a higher risk of cardioembolic stroke, systemic embolization and all cause death (3), and the cumulative incidence of thromboembolism and mortality is as high as 20% (4). Nevertheless, little is known regarding risk stratification in LVT, which warrants identification of novel prognostic biomarkers.

B-type natriuretic peptide (BNP) and N-terminal pro-B-type natriuretic peptide (NT-proBNP) are reliable diagnostic and prognostic biomarkers for HF patients (5), and are routinely used to assist diagnosis, risk stratification and treatment optimization. The peptides are mainly secreted by the left ventricle cardiomyocytes in response to ventricular stretch due to cardiovascular stress (6). High levels of BNP correlate with reduced left ventricular ejection fraction (LVEF), ventricular wall hypokinesis and akinesis (7). In addition, studies show that patients with reduced LVEF or decreased ventricular wall motion are at a higher risk of developing LVT (8–10). A study on 143 patients free of atrial fibrillation while with old myocardial infarction revealed that BNP and left ventricle segment asynergy were associated with cardioembolic stroke (11). Based on these findings, we hypothesized that the BNP level is a potential marker of LVT prognosis, and therefore retrospectively analyzed its prognostic and predictive value in patients with LVT.

## Methods

### Study Population

Patients diagnosed with LVT by transthoracic echocardiography (TTE) from November 2009 to July 2020 at the Xinqiao Hospital, Chongqing, China were enrolled for the study. The inclusion criteria were as follows: (1) LVT diagnosis by TTE, (2) age ≥ 18 years, and (3) availability of on-admission BNP or NT-pro BNP data. Patients with in-hospital mortality, cancer, non-compaction of the ventricular myocardium, tetralogy of fallot, past cardiac valve replacement, and coronary artery bypass grafting within 30 days were excluded. The study was approved by the Ethics Committee of the hospital, and was in accordance with the 1975 Declaration of Helsinki and its amendments. Given the retrospective study design, informed consent was waived.

### Data Collection and Tests

The baseline characteristics were retrieved from the medical records. LVT was screened by non-contrast TTE and contrast TTE when necessary, and identified as an echo dense mass close to a hypokinetic or akinetic myocardial segment in more than two views (12). The images were reviewed by two senior echocardiologists. BMI was calculated by dividing the body weight (kg) with the squared height (m^2^). Hypertension was diagnosed as per previous standards due to the retrospective nature of the study (13). Diabetes mellitus was diagnosed on the basis of fasting glucose >126 mg/dl or prescription of hypoglycemic drugs. Dilated cardiomyopathy was defined by the presence of left ventricular or biventricular dilatation and systolic dysfunction excluding any known cause of myocardial disease (14). Coronary heart disease (CHD) was confirmed by coronary artery computed tomography (CT), computed tomographic angiography (CTA) or percutaneous coronary angiography. All laboratory tests were conducted within 24 h of admission after overnight fasting. BNP levels were measured using the Triage Fluorescence Immunochromatographic Assay kit (Huanzhong Biotech Co. Ltd., Shijiazhuang, China) and NT-pro BNP using the ChemiLuminescent Immunoassay assay kit (Hybiome Biomedical Engineering Co. Ltd., Suzhou, China). NT-proBNP was converted to BNP using the formula BNP = NT-pro BNP/8.03 in case of atrial fibrillation, or BNP = NT-pro BNP/5.75 in the absence of atrial fibrillation (15).

### Endpoint and Follow-Up

The endpoints were all-cause mortality and systemic embolism. Survival and adverse events data were collected by office visits or telephone contacts with patients and their relatives. Loss to follow-up was defined as the lack of response to telephone contacts in addition to non-availability of follow-up medical records.

### Statistical Analysis

Continuous variables are presented as mean ± standard variation, and the categorical variables as percentages (or frequencies). Two-sample *t*-test or non-parametric Mann–Whitney *U* test were used to compare continuous variables, while categorical variables between groups were compared by Fisher exact or χ2 test. Survival curves were plotted by the Kaplan-Meier method and compared by the log-rank test. The variables with *P* < 0.10 in the univariate analysis were subjected to multivariable Cox proportional hazards regression analysis by a backward conditional method. A linear trend test was performed by entering the median value for each category of BNP quartiles. The association between BNP and all-cause mortality was evaluated using area under the receiver operating characteristic (ROC) curve (AUC). Hazard ratio (HR) and 95% confidence interval (CI) for all-cause mortality were calculated by 1 unit increase in log transformed BNP. A two-sided *P* < 0.05 was considered statistically significant. All statistical analyses were conducted using Statistical Package for the Social Sciences version 25.0 (SPSS Inc., Chicago, IL, USA).

## Results

### Baseline Characteristics

**Figure 1:**
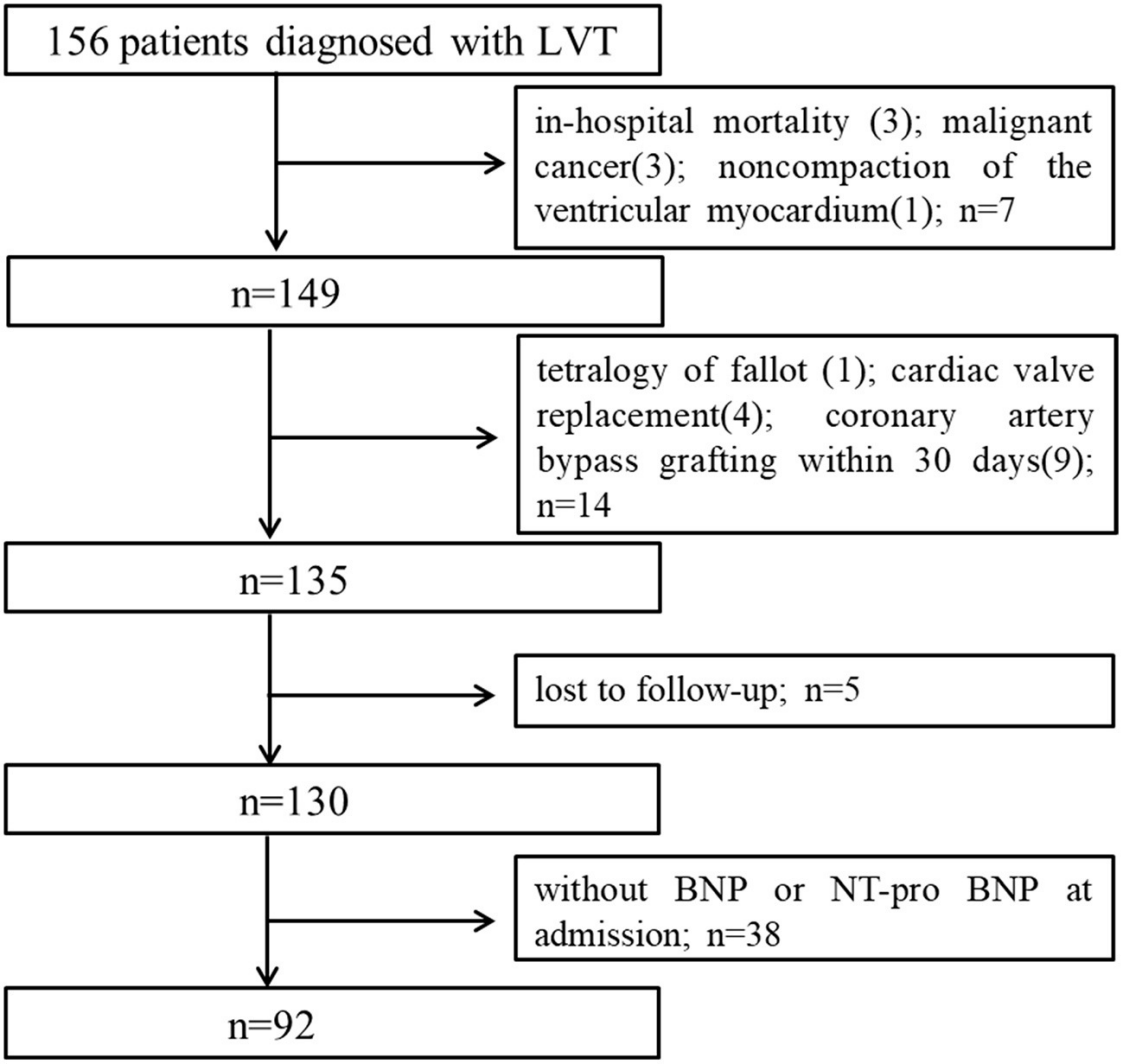
Study flow chart of subjects enrollment. LVT, left ventricular thrombus; BNP, brain natriuretic peptide.

Based on the study of 957,380 echocardiographic records, we identified 156 patients that were diagnosed with LVT by TTE, of which 92 were included in the study based on the inclusion and exclusion criteria (Figure 1). The mean age of the cohort was 56.73 ± 14.12 years, and 80.4% were male. Twenty-eight patients died during the median follow-up period of 702 days. The median BNP (1st quartile−3rd quartile) was 437.5 (112.74–1317.5). The baseline characteristics of the surviving and non-surviving patients are summarized in Table 1. The BMI, incidence of LV aneurysm, smoking, incidence of CHD, and the levels of albumin (ALB), high density lipoprotein cholesterol and triglycerides (TG) were significantly lower in the non-surviving group, whereas the incidence of dilated cardiomyopathy, white blood cell (WBC), neutrophil and monocyte counts and Log BNP level were significantly higher. No significant differences were seen in the other variables (Table 1).

**Table 1:** Baseline characteristics at admission of the LVT patients.

**Clinical characteristics**	**Total *N* = 92**	**Death group *N* = 28**	**Survival group *N* = 64**	***P-*value**
Age, years	56.73 ± 14.12	59.96 ± 16.65	55.31 ± 12.74	0.194
BMI, kg/m^2^	24.06 ± 2.96	22.92 ± 2.12	24.52 ± 3.13	0.021^*^
Gender (male)	74 (80.4)	21 (75.0)	53 (82.8)	0.385
Current smoking	52 (56.5)	10 (35.7)	42 (65.6)	0.008^*^
**Co-morbidities**				
Hypertension	40 (43.5)	12 (42.8)	28 (43.7)	0.937
Dyslipidemia	15 (16.3)	2 (7.1)	13 (20.3)	0.205
Diabetes mellitus	16 (17.4)	3 (10.7)	13 (20.3)	0.413
DCM	18 (19.6)	9 (32.1)	9 (14.1)	0.044^*^
CHD	74 (80.4)	19 (67.9)	55 (85.9)	0.044^*^
LV aneurysm	39 (42.4)	5 (17.9)	34 (53.1)	0.002^*^
Atrial fibrillation	4 (4.3)	3 (10.7)	1 (1.6)	0.154
COPD	5 (5.4)	4 (14.3)	1 (1.6)	0.983
**Laboratory findings**				
White cell count	8.13 ± 2.74	9.44 ± 3.45	7.55 ± 2.14	0.011^*^
Neutrophil count	4.94(4.0-6.51)	5.46(4.58-9.41)	4.8(3.54-5.46)	0.020^*^
Lymphcyte N	1.70 ± 0.69	1.58 ± 0.74	1.75 ± 0.66	0.257
Monocyte N	0.62 ± 0.26	0.76 ± 0.33	0.56 ± 0.21	0.002^*^
HB levels	141.34 ± 19.28	140.07 ± 15.39	141.9 ± 20.86	0.687
Platelet count	184(153-232)	200(156.5-263.5)	178(150-228)	0.256
CRP levels	5.0 (5.0-20.0)	9.35(5.43-49.48)	5.0 (5.0-20.0)	0.433
Creatinine	80.0(68.4-100.6)	82.0(67.6-106.3)	78.9(68.5-98.2)	0.788
Cysteine	1.30 ± 0.98	1.34 ± 0.58	1.29 ± 1.09	0.055
Albumin	38.86 ± 4.69	36.86 ± 3.63	39.73 ± 4.85	0.006^*^
HDL-C	0.94 ± 0.22	0.85 ± 0.26	0.98 ± 0.19	0.032^*^
TG	1.57 ± 0.87	1.22 ± 0.64	1.71 ± 0.91	0.017^*^
LDL-C	2.46 ± 0.9	2.51 ± 0.97	2.44 ± 0.88	0.864
Log BNP level	2.55 ± 0.65	2.89 ± 0.52	2.4 ± 0.65	0.001^*^
**Echocardiographic variables**				
LA diameter, mm	39.09 ± 5.21	39.88 ± 5.64	38.74 ± 5.01	0.359
LVEDd, mm	56.77 ± 9.76	59.83 ± 12.01	55.43 ± 8.36	0.086
LVEF (%)	45.98 ± 14.29	41.45 ± 16.37	47.97 ± 12.91	0.069
SV (ml)	75.1 ± 17.51	70.95 ± 20.32	76.87 ± 16.02	0.167
LV posterior thickness	10.02 ± 1.28	9.7 ± 1.29	10.16 ± 1.27	0.092
Septum thickness	10.9 ± 1.68	10.47 ± 1.79	11.09 ± 1.55	0.147

*Continuous values are documented by Mean ± SD or Median (1st quartile−3rd quartile). Categorical variables are recorded by n (percentage). BMI, body mass index; BNP, brain natriuretic peptide; CHD, coronary heart disease; COPD, chronic obstructive pulmonary disease; CRP, C-reactive protein; DCM, Dilated cardiomyopathy; HDL-C, high density lipoprotein cholesterol; LA, left atrium; LDL-C, low density lipoprotein-cholesterol; LV, left ventricular; LVEF, left ventricular ejection fraction; SV, stroke volume; LVEDd, LV end-diastolic diameter; TG, triglycerides*.

* *P < 0.05*.

### Outcomes

The all-cause mortality rate during the median follow-up duration of 702 days was 30.44% (28/92). The 1-year, 2-year, and 3-year cumulative survival rates were 85.4, 75.5, and 66.5%, respectively. Ten subjects underwent systemic embolism, of which only three survived the complication.

### COX Proportional Hazard Model

**Figure 2:**
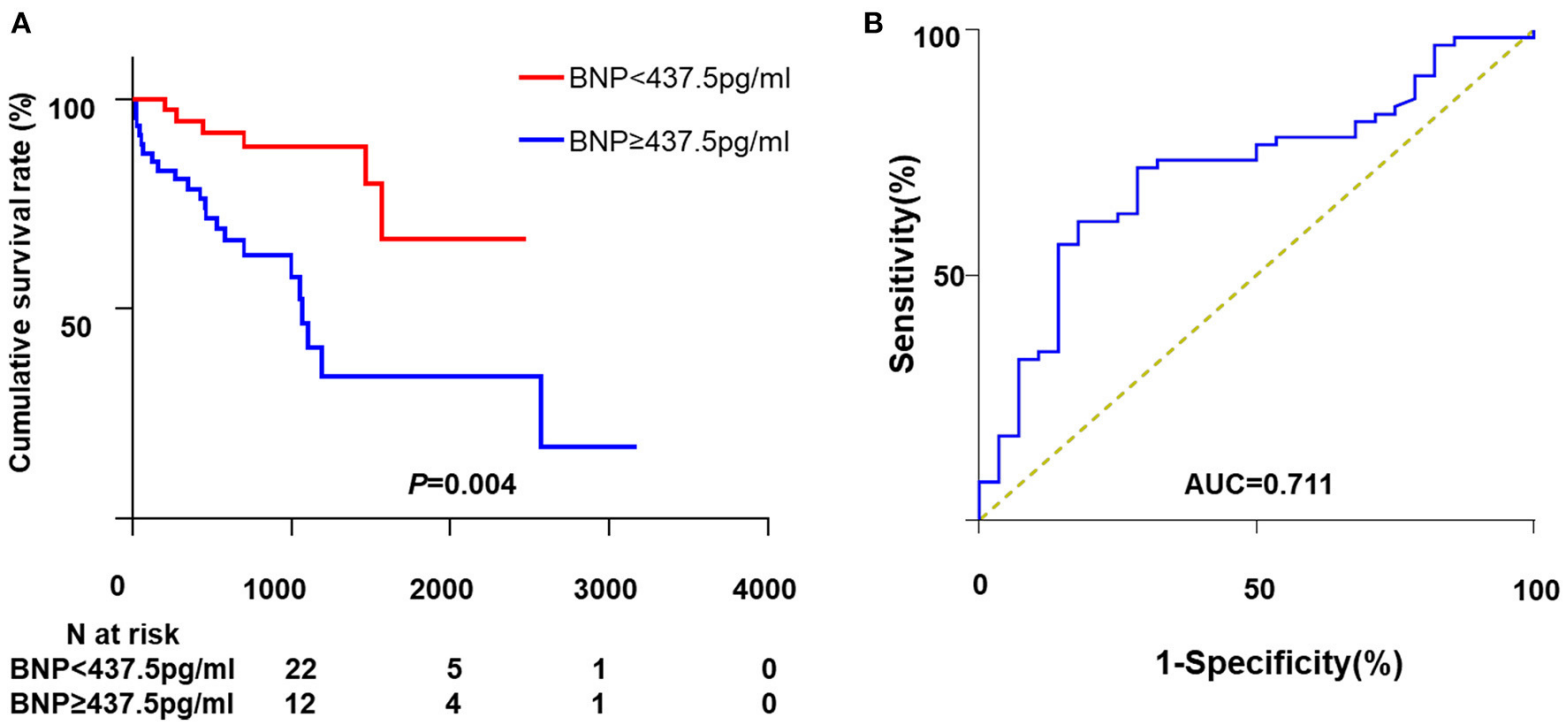
The association between BNP and all-cause mortality. **(A)** Kaplan-Meier curve analysis. **(B)** The ROC curve of BNP for all-cause mortality. ROC, receiver operating characteristic; BNP, brain natriuretic peptide; AUC, area under curve.

Univariate analysis showed that BMI, WBC, Log BNP, LV diameter, and LVEF were significantly correlated to all-cause mortality. However, only Log BNP (HR, 4.16; 95%CI, 1.81–9.56; *P* = 0.001) and BMI (HR, 0.86; 95%CI, 0.73–0.99; *P* = 0.048) were identified as the independent risk factors in the multivariate analysis (Table 2). As shown in Figure 2A, patients with BNP levels in the upper median (≥437.5 pg/ml) showed significantly higher mortality rate (*P* = 0.004) compared to those with BNP level in the lower median (<437.5 pg/ml).

**Table 2:** Backward conditional Cox univariate and multivariate analysis for all-cause mortality.

	**Univariate**	**Multivariate**			
	***P*-value**	**HR**	**Lower limit**	**Upper limit**	***P*-value**
BMI, kg/m^2^	0.031	0.86	0.73	0.99	0.048
WBC	0.007	-	-	-	-
Log BNP	0.002	4.16	1.81	9.56	0.001
LV diameter	0.011	-	-	-	-
LVEF	0.038	-	-	-	-

*BMI, body mass index; BNP, brain natriuretic peptide; LV, left ventricle; LVEF, left ventricular ejection fraction; WBC, white blood cell count*.

### ROC Curve

The ROC curve depicting the relationship between BNP and all-cause death is shown in Figure 2B. The AUC for BNP was 0.71 and the cut-off point was 667 pg/ml. The corresponding sensibility, specificity and Youden index were 0.71, 0.72, and 0.43, respectively.

### Linear Trend Test

The BNP levels were divided into four quartiles to test the prognostic value. As shown in Table 3, Model 1 was adjusted for age, gender and BMI, Model 2 for age, gender, BMI, WBC, platelet and ALB, and Model 3 for age, gender, BMI, WBC, PLT, ALB, LV diameter, LV posterior thickness, LVEF and septum thickness. BNP quartiles were significantly related to all-cause mortality in all models. The *P* for trend values for models 1, 2 and 3 were 0.005, 0.006, and 0.048, respectively, and the HRs and 95%CIs were 8.72 (1.70–102.57), 6.67 (0.84–53.14), and 13.61 (1.61–114.98), respectively (Table 3).

**Table 3:** Adjusted hazard ratios and 95% confidence interval for BNP by quartiles on all-cause mortality.

	**BNP**				
	**Q1**	**Q2**	**Q3**	**Q4**	*P* for trend^*^
	***n* = 23**	***n* = 23**	***n* = 23**	***n* = 23**	
Median pg/ml	60.8	190.0	926.0	1830.0	
Model 1	1 (reference)	2.39(0.27–21.66)	13.2(1.70–102.57)	8.72(1.70–102.57)	0.005
Model 2	1 (reference)	1.70(0.18–15.81)	9.87(1.27–77.52)	6.67(0.84–53.14)	0.006
Model 3	1 (reference)	2.39(0.27–22.25)	14.45(1.85–112.76)	13.61(1.61–114.98)	0.048

*HRs and 95%CIs were calculated by Cox proportional hazards regression models by backward conditional method;*

* *P for trend was gained by entering the median value of each category of BNP quartiles; Model 1 adjusted for age, gender, and body mass index; Model 2 adjusted for age, gender, body mass index, WBC, PLT, and ALB; Model 3 adjusted for age, gender, body mass index, WBC, PLT, ALB, LV diameter, LV posterior thickness, LVEF, and Septum thickness*.

*ALB, albumin; BNP, brain natriuretic peptide; EF, ejection fraction; LV, left ventricle; LVEF, left ventricular ejection fraction; PLT, platelet; Q, quartiles; WBC, white blood cell count*.

### Subgroup Analysis

Further, we analyzed the predicative value of BNP in the CHD subgroup. The patients with BNP levels in the upper median showed significantly higher mortality rate than that in the lower median group (*P* = 0.011). Consistently, significant differences were observed for BNP levels with regard to all-cause mortality in Kaplan–Meier analysis (*P* = 0.004; see Supplementary Material).

## Discussion

We retrospectively analyzed the predisposing factors that affect cardiovascular events and mortality in LVT patients, and found that higher BNP levels were associated with increased risk of all-cause mortality.

A previous retrospective review of more than 80,000 medical records revealed that the incidence of LVT is 7 per 10,000 patients (16). Another observational study identified 128 patients with LV thrombus from 140,636 echocardiograms (4). Consistent with these reports, the incidence of LVT was also low in our study and only 156 patients were recruited after screening more than 957,000 echocardiographic records. While PCI has markedly reduced the incidence of post-myocardial infarction LVT, cases related to heart failure have increased. Around 2/3rd of the patients in our cohort were diagnosed with CHD and the rest with dilated cardiomyopathy. The etiology of LVT patients evolves. One study showed that 80% of LVT cases are ischemic (16), and McCarthy et al. showed that *de novo* HF (38%) was more frequently associated with LVT compared to acute MI (25.9%) (4). This discrepancy may be due to the bias of small LVT population, or the different criteria utilized by the studies to analyze the precipitating factor of LVT.

Despite its low incidence rate, LVT is a lethal complication of MI or HF and is associated with high rates of systemic embolism, morbidity and mortality. We observed 10 cases of systemic embolism during the follow-up, which is similar to the reported 2–3% for PCI. Early revascularization can attenuate left ventricular dysfunction and therefore decrease the risk for LVT and associated embolism. Furthermore, anticoagulation therapy resolves the thrombus and further lowers the systemic embolism incidence to 1.9% within a year (4). In addition, Maniwa *et al*. reported a high incidence (16.3%) of systemic embolism in AMI patients with LVT (17), which can be attributed to the statistical variations in the incidence of LVT, longer follow-up period and lower primary PCI rate.

LVT patients are usually at a very high risk of developing major adverse cardiovascular events (MACE), including embolic or major bleeding complications, as well as death. A retrospective study of 159 LVT patients screened from 90,065 echocardiograms reported that MACE, stroke and all-cause mortality occurred in 37.1, 13.3, and 18.9 of the patients respectively within a median follow-up period of 632 days (18). Likewise, another study reported in-hospital mortality of 7.8% and post-discharge one-year mortality rate of 13% among LVT patients (4). Meanwhile, the one-year follow-up showed that 1.9% patients experienced strokes (4). The lower incidence of systemic embolism than mortality might hint that systemic embolism is partially accountable for the deaths. The original disease might play a more pivotal role in the mortality. In this study, we followed up the patients for 702 days and detected mortality and stroke rate of 30.44 and 11.9% (10/92), respectively. These findings collectively show the poor prognosis of LVT. Although PCI and adjunctive therapy have improved the outcome of LVT, it is crucial to identify the high-risk patients through appropriate predictive markers.

BNP is a diagnostic biomarker of HF, and its high levels are associated with poor prognosis of HF patients. MI frequently leads to ventricular dysfunction and HF, which is accompanied by BNP elevation. In our study as well, BNP was the most potent predicator of all-cause mortality of LVT patients, and elevated BNP correlated to greater risk of death. Anti-coagulation therapy is recommended to LVT patients if not contraindicated according to several guidelines. The current ESC guidelines recommend 6 months as the minimum duration of anticoagulation therapy (19). However, prolonged anti-coagulation may benefit patients with recurrent and late LVT formation. The outcomes of HF patients have improved markedly in recent years due to drugs that inhibit or even reverse cardiac remodeling. Thus, beta-blockers, angiotensin receptor neprilysin inhibitor (ARNI) and sodium-glucose cotransporter (SGLT)2 inhibitors should be initiated in patients with elevated BNP.

The study has several limitations that ought to be considered. This was a retrospective study conducted on a single center cohort, although a large number of medical records screened. Second, echocardiography was used to diagnose LVT, which might be not be as sensitive or specific as cardiac magnetic resonance imaging for detecting LVT formation. Third, the sample size was small due to the low incidence of LVT, which may have limited identification of other potential risk factors. Moreover, low sample size made us difficult to preclude all possible cofounders. The relationship between LVT and BNP was exploratory rather than causal. Finally, the results of this observational study should be considered exploratory rather than definitive.

## Conclusion

Elevated BNP is associated with a higher risk for all-cause mortality in patients with LVT, and those with BNP levels >667 pg/ml have significantly worse outcomes. Therefore, it is crucial to measure natriuretic peptide levels in the high-risk cohort.

## Data Availability Statement

The raw data supporting the conclusions of this article will be made available by the authors, without undue reservation.

## Ethics Statement

The studies involving human participants were reviewed and approved by Ethics Committee of Xinqiao Hospital. Written informed consent for participation was not required for this study in accordance with the national legislation and the institutional requirements.

## Author Contributions

ZA, ZG, and ZQ were involved in conception and design, contributed to interpretation of data, involved in statistical analysis, and helped in drafting of the manuscript. ZA, ZG, CH, LW, and LZ helped in acquisition of data. CL, RR, and SG contributed to review of ultrasonic data. ZA, ZG, CH, and ZQ were involved in final revision of manuscript. ZQ helped in study supervision. All authors contributed to critical revision of the manuscript for important intellectual content. All authors read and approved the final manuscript.

## Conflict of Interest

The authors declare that the research was conducted in the absence of any commercial or financial relationships that could be construed as a potential conflict of interest.

## Abbreviations

ALB: albumin
BMI: body mass index
BNP: brain natriuretic peptide
CHD: coronary heart disease
COPD: chronic obstructive pulmonary disease
CRP: C-reactive protein
CTA: computed tomographic angiography
HDL: high density lipoprotein
LA: left atrium
LVEF: left ventricular ejection fraction
LDL-C: low density lipoprotein- cholesterol
LVT: left ventricular thrombus
PCI: percutaneous coronary intervention
PLT: platelet
SV: stroke volume
TG: triglycerides
WBC: white blood cell count.

## References

[1] Delewi R Nijveldt R Hirsch A Marcu CB Robbers L Hassell ME. Left ventricular thrombus formation after acute myocardial infarction as assessed by cardiovascular magnetic resonance imaging. Eur J Radiol. (2012) 81:3900–4. 10.1016/j.ejrad.2012.06.029 22995173

[2] Habash F Vallurupalli S. Challenges in management of left ventricular thrombus. Ther Adv Cardiovasc Dis. (2017) 11:203–13. 10.1177/1753944717711139 28589748 PMC5933579

[3] Zhang Q Wang CM Shi ST Chen H Zhou YJ. Relationship of left ventricular thrombus formation and adverse outcomes in acute anterior myocardial infarction in patients treated with primary percutaneous coronary intervention. Clin Cardiol. (2019) 42:69–75. 10.1002/clc.23106 30367476 PMC6436520

[4] McCarthy CP Murphy S Venkateswaran RV Singh A Chang LL Joice MG. Left ventricular thrombus: contemporary etiologies, treatment strategies, and outcomes. J Am Coll Cardiol. (2019) 73:2007–9. 10.1016/j.jacc.2019.01.031 30846340

[5] Richards AM. N-Terminal B-type Natriuretic Peptide in Heart Failure. Heart Fail Clin. (2018) 14:27–39. 10.1016/j.hfc.2017.08.004 29153198

[6] Yancy CW Jessup M Bozkurt B Butler J Casey DE Jr Colvin MM. 2017 ACC/AHA/HFSA Focused Update of the 2013 ACCF/AHA Guideline for the Management of Heart Failure: a Report of the American College of Cardiology/American Heart Association Task Force on Clinical Practice Guidelines and the Heart Failure Society of America. Circulation. (2017) 136:e137-e161. 10.1161/cir.0000000000000509 28455343

[7] Chen AA Wood MJ Krauser DG Baggish AL Tung R Anwaruddin S. NT-proBNP levels, echocardiographic findings, and outcomes in breathless patients: results from the ProBNP Investigation of Dyspnoea in the Emergency Department (PRIDE) echocardiographic substudy. Eur Heart J. (2006) 27:839–45. 10.1093/eurheartj/ehi811 16510467

[8] Gianstefani S Douiri A Delithanasis I Rogers T Sen A Kalra S. Incidence and predictors of early left ventricular thrombus after ST-elevation myocardial infarction in the contemporary era of primary percutaneous coronary intervention. Am J Cardiol. (2014) 113:1111–6. 10.1016/j.amjcard.2013.12.015 24485697

[9] Shacham Y Leshem-Rubinow E Ben Assa E Rogowski O Topilsky Y Roth A. Frequency and correlates of early left ventricular thrombus formation following anterior wall acute myocardial infarction treated with primary percutaneous coronary intervention. Am J Cardiol. (2013) 111:667–70. 10.1016/j.amjcard.2012.11.016 23261006

[10] Bakalli A. Georgievska-Ismail L. Koçinaj D. Musliu N. Krasniqi A. Pllana E. (2013). Prevalence of left chamber cardiac thrombi in patients with dilated left ventricle at sinus rhythm: the role of transesophageal echocardiography. J Clin Ultrasound 41(1), 38–45. 10.1002/jcu.21953 22729833

[11] Hosomi N Yoshimoto T Kanaya Y Neshige S Hara N Himeno T. Brain natriuretic peptide and particular left ventricle segment asynergy associated with cardioembolic stroke from old myocardial infarction. J Stroke Cerebrovasc Dis. (2016) 25:1165–71. 10.1016/j.jstrokecerebrovasdis.2016.02.003 26922130

[12] Stratton JR Lighty GW Jr Pearlman AS Ritchie JL. Detection of left ventricular thrombus by two-dimensional echocardiography: sensitivity, specificity, and causes of uncertainty. Circulation. (1982) 66:156–66. 10.1161/01.cir.66.1.156 7083502

[13] Williams B Mancia G Spiering W Agabiti Rosei E Azizi M Burnier M. 2018 ESC/ESH Guidelines for the management of arterial hypertension. Eur Heart J. (2018) 39:3021–104. 10.1093/eurheartj/ehy339 30165516

[14] McNally EM Mestroni L. Dilated cardiomyopathy: genetic determinants and mechanisms. Circ Res. (2017) 121:731–48. 10.1161/circresaha.116.309396 28912180 PMC5626020

[15] Rorth R Jhund PS Yilmaz MB Kristensen SL Welsh P Desai AS. Comparison of BNP and NT-proBNP in patients with heart failure and reduced ejection fraction. Circ Heart Fail. (2020) 13:e006541. 10.1161/CIRCHEARTFAILURE.119.006541 32065760

[16] Lee JM Park JJ Jung HW Cho YS Oh IY Yoon CH. Left ventricular thrombus and subsequent thromboembolism, comparison of anticoagulation, surgical removal, and antiplatelet agents. J Atheroscler Thromb. (2013) 20:73–93. 10.5551/jat.13540 22986555

[17] Maniwa N Fujino M Nakai M Nishimura K Miyamoto Y Kataoka Y. Anticoagulation combined with antiplatelet therapy in patients with left ventricular thrombus after first acute myocardial infarction. Eur Heart J. (2018) 39:201–8. 10.1093/eurheartj/ehx551 29029233

[18] Lattuca B Bouziri N Kerneis M Portal JJ Zhou J Hauguel-Moreau M. Antithrombotic Therapy for patients with left ventricular mural thrombus. J Am Coll Cardiol. (2020) 75:1676–85. 10.1016/j.jacc.2020.01.057 32273033

[19] Ibanez B James S Agewall S Antunes MJ Bucciarelli-Ducci C Bueno H. 2017 ESC Guidelines for the management of acute myocardial infarction in patients presenting with ST-segment elevation. Eur Heart J. (2018) 39:119–77. 10.1093/eurheartj/ehx393 28886621

