# Supplementary material for: Prognostic Significance of B-Type Natriuretic Peptide in Patients With Left Ventricular Thrombus
Source: Front Cardiovasc Med. 2021 Apr 29;8:667908. doi: 10.3389/fcvm.2021.667908 (PMC8116499; doi:10.3389/fcvm.2021.667908)
Supplement: Supplementary file 1 [file Data_Sheet_1.docx]

|  | [Univariate](https://cn.bing.com/dict/search?q=univariate&FORM=BDVSP6&mkt=zh-cn) | [Multivariate](https://cn.bing.com/dict/search?q=multivariate&FORM=BDVSP6&mkt=zh-cn) |  |  |  |
| --- | --- | --- | --- | --- | --- |
|  | *P* value | HR | Lower limit | Upper limit | *P* value |
| Age | 0.097 | - | - | - | - |
| WBC | 0.011 | - | - | - | - |
| PLT | 0.020 | - | - | - | - |
| BNP>median | 0.011 | 4.27 | 1.4 | 13.01 | 0.011 |

**Supplementary Data**

**Supplementary Table 1 Backward Conditional Cox** [**Univariate**](https://cn.bing.com/dict/search?q=univariate&FORM=BDVSP6&mkt=zh-cn) **and Multivariate Analysis for All-cause Mortality in CHD patients**

*WBC: white blood cell count; PLT: platelet; BNP: brain natriuretic peptide; CHD: coronary heart disease;*


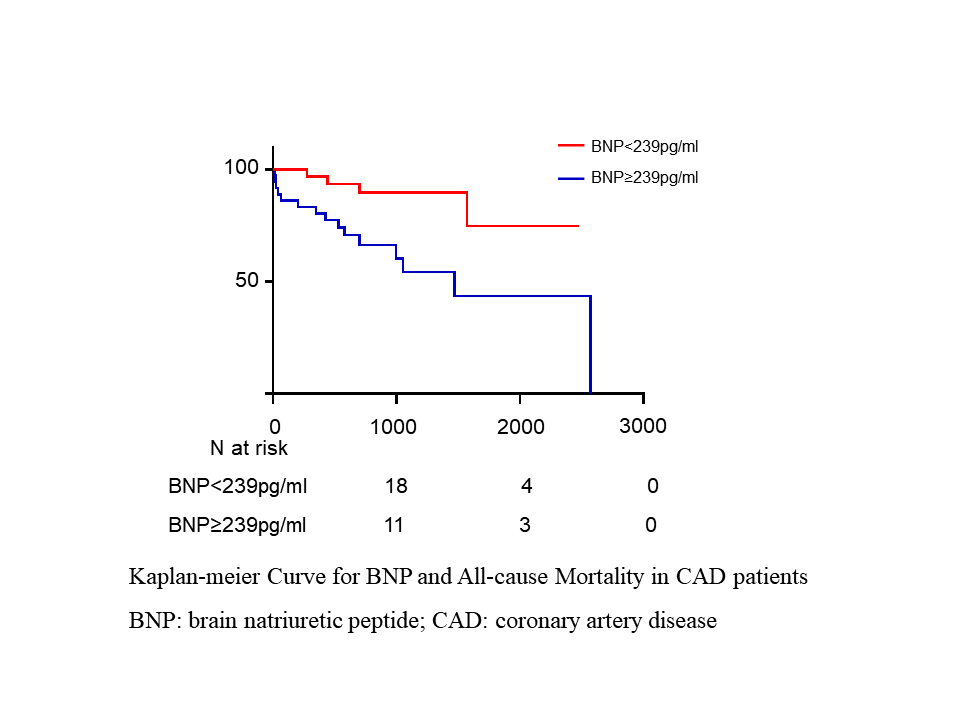
**Supplementary Figure 1** Kaplan-meier Curve for BNP and All-cause Mortality in CHD patients.

BNP: brain natriuretic peptide; CHD: coronary heart disease
